# Effect of Coronavirus Disease 2019 (COVID-19) on Elite Spanish Student-Athletes’ Perception of the Dual Career

**DOI:** 10.3389/fpsyg.2020.620042

**Published:** 2020-12-21

**Authors:** Lucia Abenza-Cano, Alejandro Leiva-Arcas, Raquel Vaquero-Cristóbal, Juan Alfonso García-Roca, Lourdes Meroño, Antonio Sánchez-Pato

**Affiliations:** Olympic Studies Center, Faculty of Sport, Catholic University of Murcia, Murcia, Spain

**Keywords:** academic career, Coronavirus (COVID-19), dual career, sport career, sport tutor, student-athlete, university

## Abstract

The aim of the present research was to assess elite student-athletes’ perception of the dual career during the lockdown caused by the coronavirus disease 2019 (COVID-19) pandemic, compared with a group of elite student-athletes who could develop their dual career under normal conditions. A total of 150 elite athletes who were also undergraduate or postgraduate students self-completed the “Perceptions of dual career student-athletes (ESTPORT)” questionnaire. From them, 78 did it during the mandatory lockdown period due to the state of emergency caused by COVID-19 (COVID-19 group) and 72 completed it in the previous year to Rio 2016 Olympic Games (control group). The COVID-19 group was found to spend a significantly higher number of hours per week studying, while no significant differences were observed between groups in any training time variable. Student-athletes of the COVID-19 group showed better perception of whether their sport career could help them cope with their academic career and better general perception of remote learning and the use of tasks and videoconferencing as learning support tools. A lower percentage of athletes of the COVID-19 group than of the control group wished to continue with their sport career once they finished their studies. To conclude, student-athletes of the COVID-19 group show adaptations with regard to the organization of their studies and the importance they give to them and to the services provided by dual-career programs, compared with student-athletes from an ordinary pre-Olympic year. In general, student-athletes’ perception of the dual career is very positive.

## Introduction

Spain has been one of the most affected countries by the pandemic (according to the WHO, on March 14, 2020, there were 4,231 cases and had been 120 deaths, and by the end of the lockdown, on May 3, 216,582 cases and 25,100 deaths) (World Health Organization, 2020). The state of emergency declared in Spain on March 14, 2020, due to the health crisis derived from the coronavirus disease 2019 (COVID-19) pandemic entailed home lockdown, which was mandatory except to perform activities considered essential (Agencia Estatal Boletín Oficial del Estado, 2020b). This fact had a big impact on the daily life (changes in daily routines and cancelation of important activities) and psychological factors (increased anxiety, stress, and depression, among others) of the Spanish population (Rodríguez-Rey et al., 2020).

From that moment, face-to-face lessons were suspended in Spanish universities, and teaching and evaluation have been adapted to remote or online mode, provided it was feasible (Odriozola-González et al., 2020). In this exceptional scenario, the Spanish university system, with the effort of the whole university community and the institutions, provided a responsible, prompt, and agile response in order to guarantee the continuation of the academic activities by adapting to remote methods (Torrecillas, 2020). The different bodies with competence in high education in Spain in this unique situation agreed on the following academic criteria: (1) students should not miss the academic year due to this crisis, or would not be overcharged due to the measures adopted related to teaching; (2) administration, universities, and agencies would join forces in order to ensure the academic quality of the training received by students during the academic year 2019–2020; and (3) universities would autonomously manage and develop their official degrees, and the competent authorities (self-governed regions, with collaboration of the Ministry of Universities) would supervise the process in order to guarantee the system’s quality standards (Conferencia General de Poliìtica Universitaria, 2020).

This situation has affected the normal development of the dual career. The concept of dual career refers to the combination of an athletic career with education and/or occupation (Geraniosova and Ronkainen, 2015). Educational models that promote the dual career are based on the human right to education and guarantee that elite athletes can train and compete and at the same time develop their academic career (Sánchez-Pato et al., 2017). For an athlete, pursuing education while competing in high-performance sport can be a challenging task. The increasing demands on athletic performance in elite sports place high pressure on athletes, who may feel forced to choose between maximizing their athletic potential or obtaining a satisfying education for a post-athletic career (Lavallee and Wylleman, 2000). As stated by Geraniosova and Ronkainen (2015), this situation may lead to premature discontinuation of the athletic career due to prioritization of education (Amara et al., 2004) or, by contrast, to lower investment in education due to exclusive focus on achieving athletic success (Aries et al., 2004).

It is essential to keep a holistic and systematic approach that can appropriately adapt to the essence of education as an organic, complex, circular, dynamic, and open process, promoting the participation of all agents in order to trigger improvements in the education, sport, professional, and personal systems (Isidori, 2016). The student-athlete is the leading role in this scenario where sport is the main life axis and an athletic identity based on success determines a personality that represents the values and is the source of affective and interpersonal relationships (Lally and Kerr, 2005). This identity must be preserved and, at the same time, developed in such a manner that the athlete accepts, with responsibility, the student role that will allow them to broaden their personal knowledge and to work on their own future through the acquisition of specific skills (Migliorati et al., 2016).

Some universities facilitate this process through the creation of a key role to ensure dual-career efficiency: the sport tutor. From this perspective, it seems necessary to offer a personalized tutorship model using a clear pedagogic approach in which sport must be always connected to comprehensive education. Thus, there must be a very close relationship between sport and continuous learning, based on mutual acknowledgment (Isidori, 2016). In this regard, the sport tutor plays the role of an academic guide, encouraging the achievement of good academic results, and of a mediator among student-athletes, the university, and the sport organizations. Furthermore, they plan the training and studying schedules and monitor and support the academic career through e-learning and other online support tools, especially during competition periods when the student-athlete cannot attend university (Isidori, 2016).

Elite student-athletes, who work on their sport and academic careers at the same time, have needed to adapt both their training and studies due to the lockdown caused by COVID-19. Under normal conditions, student-athletes make an effort to perform both activities simultaneously, organizing and optimizing their time with the help of their tutor. In an extraordinary situation like the one arising during the COVID-19 pandemic, student-athletes stopped attending their training centers and needed to adapt to home training with very limited resources and facilities (Toresdahl and Asif, 2020). All sport events and on-site training were postponed or canceled, leading to economic loss (Jiménez-Gutiérrez et al., 2020). In line with this, the report created by the Association for Spanish Sport (Asociación del Deporte Español, ADESP) together with Active Spain Federation (Fundación España Activa) and the High Council for Sport (CSD) has yielded very relevant results as regards the impact that the health crisis has had on the Spanish sport system. The estimated loss for 2020 is 4.6 billion Euro, meaning 38.5% of the revenue expected by the participating organizations in 2020. According to this report, COVID-19 has also caused a decrease of 31% in athletes’ income. The unemployment rate within the sector has raised by the same percentage (Jiménez-Gutiérrez et al., 2020). Besides this uncertainty, on March 24, 2020, the International Olympic Committee announced that Tokyo Olympic Games (OG) would be postponed 1 year, until summer 2021, because of the pandemic (International Olympic Committe, 2020). This unprecedented decision left elite athletes in a standby situation as regards the training plan followed during the previous 4 years. These athletes resumed training individually, after a 7-week lockdown, on Monday, May 4 (beginning of lockdown ease in stages), when basic training was allowed again as well for professional leagues and high-level athletes (Agencia Estatal Boletín Oficial del Estado, 2020a).

Nevertheless, the lockdown gave them the opportunity to focus on other tasks, such us continuing with their studies online, since Spanish universities switched to this more accessible and flexible modality (Odriozola-González et al., 2020), which are key features in the success of the dual career (Sánchez-Pato et al., 2017).

Studies on the impact of COVID-19 on sport have started to emerge in different fields, especially in those related to the negative effects on elite athletes’ performance and physical fitness after the pandemic (Baggish et al., 2020; Dores and Cardim, 2020), athletes’ psychological aspects (Sarto et al., 2020), financial and structural aspects of sport organizations (Drewes et al., 2020), or the consequences of the cancelation or postponement of sport events such as the Tokyo 2020 OG (Gallego et al., 2020). Nevertheless, beyond sport itself, there is a lack of research on the effects of COVID-19 on complementary elements of elite athletes’ life like, for example, their student role. Up to date, no study has been found to address this topic. Therefore, the aim of the present research was to assess elite student-athletes’ perception of the dual career during the lockdown caused by the COVID-19 pandemic, compared with a group of elite student-athletes who could develop their dual career under normal conditions, both in pre-Olympic years.

## Materials and Methods

### Design and Participants

A descriptive, cross-sectional study design with non-probability-based sampling was used. The sample was selected based on convenience. It comprised 150 elite Spanish student-athletes (50% male and 50% female), of mean age 25.29 years (*SD* = 4.67). All of them competed or were preparing to compete at the OG that would be held the following year. They had all previously participated in international competitions. Participants belonged to two different groups that remained academically active during the academic years 2015–2016 (control group; *n* = 72), previous to Rio 2016 OG, and 2019–2020 (COVID-19 group; *n* = 78), previous to Tokyo 2020 OG, within the dual-career university program (Table 1). The inclusion criteria were as follows: (a) to be enrolled on any university degree or master’s studies within a dual-career university program; (b) to be considered a high-level athlete according to the Spanish High for Sport and to be included in the list published in the national official bulletin (Boletín Oficial del Estado, BOE); (c) to have participated in international competitions; and (d) to have participated in the previous OG or to be eligible to participate in the upcoming OG. The sample consisted of undergraduate (*n* = 120; 80.00%) or postgraduate (*n* = 30; 20.00%) students and, at the same time, elite athletes of individual (*n* = 97; 64.66%) or team sports (*n* = 35.33%) who were in different stages of their sport career: beginning (*n* = 43; 28.66%), top (*n* = 78; 52.00%), or end (*n* = 29; 19.33%).

**TABLE 1:** Descriptive statistics of student-athletes in normal pre-Olympic year (control group) and student-athletes in COVID-19 pre-Olympic year (COVID-19 group).

		**Control group**	**COVID-19 group**	**Differences between groups**
Age (years)		25.40 ± 4.37	25.18 ± 4.97	*t* = 0.29; *p* = 0.77; *d* = 0.05; 95% CI = −1.30 to 1.74
Sex (*n*,%)	Male	38 (52.8%)	37 (47.4%)	χ^2^ = 0.44; *p* = 0.51
	Female	34 (47.2%)	41 (52.6%)	
Type of sport	Individual	50 (69.4%)	47 (60.3%)	χ^2^ = 1.38; *p* = 0.24
	Team	22 (30.6%)	31 (39.7%)	
Self-consideration of spor performance level	Amateur	40 (55.6%)	36 (46.2%)	χ^2^ = 1.44; *p* = 0.49
	Semiprofessional	29 (40.3%)	39 (50.0%)	
	Professional	3 (4.2%)	3 (3.8%)	
Self-consideration of stage in sport career	Beginning of high-level competition	15 (20.8%)	28 (35.9%)	χ^2^ = 5.29; *p* = 0.07
	Top performance in high-level competition	44 (61.1%)	34 (43.6%)	
	End of high-level competition	13 (18.1%)	16 (20.5%)	
Ongoing studies	Degree	56 (77.8%)	64 (82.1%)	χ^2^ = 0.43; *p* = 0.51
	Master’s degree	16 (22.2%)	14 (17.9%)	
Employed	Yes	25 (34.7%)	30 (38.5%)	χ^2^ = 0.22; *p* = 0.63
	No	47 (65.3%)	48 (61.5%)	
Study completion pace (years/level)		1.88 ± 0.89	1.87 ± 1.14	*t* = 0.02; *p* = 0.98; *d* = 0.01; 95% CI = −0.33 to 0.33
Self-perception	Athlete-student	18 (25.0%)	17 (21.8%)	χ^2^ = 0.21; *p* = 0.64
	Student-athlete	54 (75.0%)	61 (78.2%)	
Priority	Studies	28 (38.9%)	41 (52.6%)	χ^2^ = 2.82; *p* = 0.09
	Athletic career	44 (61.1%)	37 (47.4%)	
Hours per week spent on studying		9.87 ± 8.59	12.93 ± 8.60	*t* = −2.15; *p* = 0.03; *d* = 0.36; 95% CI = −1.58 to 3.32
Training sessions per week	Between 1 and 5	13 (18.1%)	19 (24.4%)	χ^2^ = 1.51; *p* = 0.47
	Between 6 and 10	38 (52.8%)	42 (53.8%)	
	More than 10	21 (29.2%)	17 (21.8%)	
Training hours per week	Fewer than 5	0 (0.0%)	1 (1.3%)	χ^2^ = 3.78; *p* = 0.44
	Between 5 and 10	7 (9.7%)	15 (19.2%)	
	Between 11 and 15	18 (25.0%)	17 (21.8%)	
	Between 16 and 20	20 (27.8%)	20 (25.6%)	
	More than 20	27 (37.5%)	25 (32.1%)	

### Sample Size

The calculations to establish the sample size were performed using RStudio 3.15.0 software. Significance level was set at *a* = 0.01. The standard deviation (SD) was determined based on a previous study (Sánchez-Pato et al., 2016). With an estimated error (*d*) of 0.33, a valid sample size for a 99% confidence interval (CI) was found to be 72 for each group.

### Data Collection

The perceptions of university student-athletes as regards their dual career were analyzed using a simple version of the questionnaire “Perceptions of dual career student-athletes (ESTPORT)” (Sánchez-Pato et al., 2016; Gavala-González et al., 2019). The instrument was composed of 84 items, with various question types (Likert scale, multiple choice, short answer). Most questionnaire items used a Likert-type scale ranging from 1 (“strongly disagree”) to 5 (“strongly agree”). This assessment tool was divided into two categories: (a) sociodemographic and context variables (14 items) and (b) dual-career aspects (70 items). The items about dual career comprised three subscales: “academic career” (38 items), “sport career” (15 items), and “sport tutor” (11 items). In addition, it included some questions about the dual career (six items). The results revealed high internal consistency of the questionnaire, since Cronbach’s alpha coefficients were higher than 0.70 in the three dimensions: academic career (alpha = 0.81), sport career (alpha = 0.73), and sport tutor (alpha = 0.93), which are above the lower limit accepted as reliable (Corbetta, 2007; Sánchez-Pato et al., 2016).

### Procedure

The study obtained the approval from the ethics committee of the authors’ university (code: 19/6/2015). The student-athletes were contacted through the university sport services and the Spanish Olympic Committee. Participants were contacted *via* email to participate in the study. The student-athletes were informed about the aims and procedure of the study through an informed consent form. The student-athletes completed the questionnaire anonymously and individually at home, without being under academic or competition pressure, without the presence of their coach or professors. After signing the informed consent, they could start completing the questionnaire. The participants did not receive any additional explanation about the purpose of the questionnaire apart from that contained in the questionnaire itself. The questionnaire was accessed online using Google Forms^®^. The participants completed it during 20–30 min. The COVID-19 group self-completed the questionnaire during the week before the lockdown ease started (seventh week of lockdown, between April 27 and May 01, 2020), when individual outdoor training was allowed again.

### Statistical Analysis

The scale internal consistency values were acceptable (Streiner, 2003). After assessing variable normality through the Kolmogorov–Smirnov test, homogeneity through Levene’s test, and sphericity through the Mauchly test, a descriptive analysis was conducted for the quantitative (means and standard deviations) and qualitative variables (counts and percentages). The differences between the two groups (COVID-19 and control groups) in the continuous variables were determined using unpaired *t*-tests. The effect size was calculated, defined as low (*r* = 0.10), moderate (*r* = 0.30), high (*r* = 0.50), or very high (*r* = 0.70) (Cohen, 1988). Chi-square analyses were used to analyze differences between groups in the categorical variables. Cramer’s *V* was used for *post hoc* comparison of 2 × 2 tables and the contingency coefficient was used for 2 × *n* tables, obtaining the value of the statistic and the *p*-value. The maximum expected value was 0.707; *r* < 0.3 indicated low association, *r* between 0.3 and 0.5 indicated moderate association, and *r* > 0.5 meant high association. The statistical analysis was performed using the statistical package SPSS 21.0 for Windows. An error of *p* ≤ 0.05 was established.

## Results

Table 1 contains the results of the descriptive variables for both groups. No statistically significant differences were found between groups (*p* > 0.05). The COVID-19 group was found to spend a significantly higher number of hours per week studying, with a moderate effect (*p* = 0.03), while no significant differences were observed between groups in the number of training sessions or training hours per week (*p* > 0.05).

Table 2 shows the athletes’ perceptions and opinions about the dual career. The COVID-19 group showed better perception of whether their sport career could help them cope with their academic career (*p* = 0.03) and better general perception of remote learning (*p* < 0.001). No significant differences were detected in the perception of interference of studies with athletic performance or vice versa, or in the perception of the sport career–academic career balance (*p* > 0.05). All participants perceived certain interferences between both spheres and admitted that it was not easy to achieve good balance.

**TABLE 2:** Comparison of the perception of the dual career, according to the variables studied, between student-athletes in normal pre-Olympic year (control group) and student-athletes in COVID-19 pre-Olympic year (COVID-19 group).

		**Control group**	**COVID-19 group**	**Differences between groups**
Interference of studies with athletic performance	Yes	33(45.8%)	40(51.3%)	χ^2^ = 0.44; *p* = 0.50
	No	39(54.2%)	38(48.7%)	
Interference of athletic performance with studies	Yes	49(68.1%)	57(73.1%)	χ^2^ = 0.45; *p* = 0.50
	No	23(31.9%)	21(26.9%)	
My sport career helps cope with my studies	Strongly agree	11(15.3%)	15(19.2%)	χ^2^ = 10.78; *p* = 0.03
	Agree	15(20.8%)	28(35.9%)	
	Neither agree nor disagree	28(38.9%)	21(26.9%)	
	Disagree	7(9.7%)	11(14.1%)	
	Strongly disagree	11(15.3%)	3(3.8%)	
Balance between my sport career and academic career	Very easy	0(0.0%)	2(2.6%)	χ^2^ = 4.93; *p* = 0.29
	Easy	14(19.4%)	18(23.1%)	
	Neither easy nor difficult	22(30.6%)	26(33.3%)	
	Difficult	25(34.7%)	27(34.6%)	
	Very difficult	11(15.3%)	5(6.4%)	
I encounter barriers to achieve good balance between my sport career and academic career	Strongly agree	2(2.8%)	0(0.0%)	χ^2^ = 4.10; *p* = 0.39
	Agree	8(11.1%)	5(6.4%)	
	Neither agree nor disagree	25(34.7%)	27(34.6%)	
	Disagree	30(41.7%)	34(43.6%)	
	Strongly disagree	7(9.7%)	12(15.4%)	
Perception of sport monitoring services as part of the university dual career	Very high	14(19.4%)	19(24.4%)	χ^2^ = 4.79; *p* = 0.31
	High	17(23.6%)	28(35.9%)	
	Neutral	24(33.3%)	17(21.8%)	
	Somewhat high	10(13.9%)	9(11.5%)	
	Not high at all	7(9.7%)	5(6.4%)	
Perception of flexible curriculum	Very high	23(31.9%)	34(43.6%)	χ^2^ = 7.40; *p* = 0.12
	High	18(25.0%)	21(26.9%)	
	Neutral	20(27.8%)	20(25.6%)	
	Somewhat high	8(11.1%)	3(3.8%)	
		3(4.2%)	0(0.0%)	
Perception of remote learning	Very high	29(40.3%)	26(33.3%)	χ^2^ = 20.16; *p* < 0.001
	High	14(19.4%)	38(48.7%)	
	Neutral	11(15.3%)	14(17.99%)	
	Somewhat high	10(13.9%)	3(3.8%)	
	Not high at all	5(6.9%)	0(0.0%)	
Perception of the sport tutor services as part of the university dual career	Very high	30(41.7%)	30(38.5%)	χ^2^ = 5.62; *p* = 0.23
	High	13(18.1%)	22(28.2%)	
	Neutral	10(13.9%)	15(19.2%)	
	Somewhat high	8(11.1%)	6(7.7%)	
	Not high at all	11(15.3%)	5(6.4%)	
Perception of the need for sport tutor services as part of the university dual career	Strongly disagree	8(11.8%)	15(20.5%)	χ^2^ = 4.43; *p* = 0.35
	Disagree	2(2.9%)	5(6.8%)	
	Neither disagree nor agree	13(19.1%)	13(17.8%)	
	Agree	26(38.2%)	19(26.0%)	
	Strongly agree	19(27.9%)	21(28.8%)	
Use of the virtual campus as learning tool	Always	43(60.6%)	56(71.8%)	χ^2^ = 6.45; *p* = 0.17
	Almost always	11(15.5%)	13(16.7%)	
	Sometimes	10(14.1%)	8(10.3%)	
	Almost never	2(2.8%)	0(0.0%)	
	Never	5(7.0%)	1(1.3%)	
Use of online fora as learning tool	Always	6(8.3%)	10(13.0%)	χ^2^ = 6.54; *p* = 0.16
	Almost always	9(12.5%)	8(10.4%)	
	Sometimes	6(8.3%)	16(20.8%)	
	Almost never	11(15.3%)	7(9.1%)	
	Never	40(55.6%)	36(46.8%)	
Use of online tasks as learning tool	Always	23(31.9%)	40(52.6%)	χ^2^ = 19.46; *p* = 0.001
	Almost always	16(22.2%)	18(23.7%)	
	Sometimes	12(16.7%)	15(19.7%)	
	Almost never	5(6.9%)	2(2.6%)	
	Never	16(22.2%)	1(1.3%)	
Use of online chat as learning tool	Always	4(5.6%)	4(5.3%)	χ^2^ = 3.41; *p* = 0.49
	Almost always	4(5.6%)	6(7.9%)	
	Sometimes	4(5.6%)	9(11.8%)	
	Almost never	10(13.9%)	14(18.4%)	
	Never	50(69.4%)	43(56.6%)	
Use of videoconferencing as learning tool	Always	1(1.4%)	19(25.0%)	χ^2^ = 50.21; *p* < 0.001
	Almost always	1(1.4%)	10(13.2%)	
	Sometimes	9(12.5%)	18(23.7%)	
	Almost never	5(6.9%)	12(15.8%)	
	Never	56(77.8%)	14(22.4%)	
Use of Facebook as learning tool	Always	2(2.9%)	6(7.8%)	χ^2^ = 5.41; *p* = 0.25
	Almost always	2(2.9%)	3(3.9%)	
	Sometimes	5(7.4%)	11(14.3%)	
	Almost never	5(7.4%)	9(11.7%)	
	Never	54(79.4%)	48(62.3%)	
Use of Twitter as learning tool	Always	6(8.5%)	11(14.3%)	χ^2^ = 2.39; *p* = 0.66
	Almost always	3(4.2%)	6(7.8%)	
	Sometimes	8(11.3%)	8(10.4%)	
	Almost never	9(12.7%)	10(13.0%)	
	Never	45(63.4%)	42(54.5%)	

However, the majority of athletes from both groups did not perceive barriers that could hinder their success in the dual career. In general, they showed positive perception of the flexible curriculum, sport monitoring of the dual career by the university, the role of the sport tutor, and the services provided by the sport tutor, with no differences between groups in any of these variables (*p* > 0.05).

With regard to the use of learning support tools, the COVID-19 group considered the tools tasks (*p* = 0.001) and videoconferencing (*p* < 0.001) to be more important than the control group. Both groups showed positive perception of the use of the virtual campus as a learning support tool, while they did not consider the use of fora, chat, Facebook, or Twitter relevant, with no significant differences in the use of these tools (*p* > 0.05).

Table 3 shows the athletes’ expectations after finishing their university studies. A lower percentage of athletes of the COVID-19 group than of the control group wished to continue with their sport career once they finished their studies (*p* = 0.01). By contrast, there were no significant differences in the rest of the items related to their expectations after completing their studies (*p* > 0.05).

**TABLE 3:** Comparison of expectations between student-athletes in normal pre-Olympic year (control group) and student-athletes in COVID-19 pre-Olympic year (COVID-19 group).

		**Control group**	**COVID-19 group**	**Differences between groups**
To continue with further studies	Yes	36 (50.0%)	38 (48.7%)	χ^2^ = 0.02; *p* = 0.87
	No	36 (50.0%)	40 (51.3%)	
To work in the area of university studies	Yes	56 (77.8%)	61 (78.2%)	χ^2^ = 0.00; *p* = 0.95
	No	16 (22.2%)	17 (21.8%)	
To work in a different area from university studies	Yes	3 (4.2%)	1 (1.3%)	χ^2^ = 1.20; *p* = 0.273
	No	69 (95.8%)	77 (98.7%)	
To continue with sport career	Yes	52 (72.2%)	40 (51.3%)	χ^2^ = 6.92; *p* = 0.01
	No	20 (27.8%)	38 (48.7%)	
To continue being involved in sport	Yes	61 (84.7%)	66 (84.6%)	χ^2^ = 0.00; *p* = 0.99
	No	11 (15.3%)	12 (15.4%)	
To live on my savings	Yes	1 (1.4%)	3 (3.8%)	χ^2^ = 0.87; *p* = 0.35
	No	71 (98.6%)	75 (96.2%)	

## Discussion

The aim of the present research was to assess elite student-athletes’ perception of the dual career during the lockdown caused by the COVID-19 pandemic, compared with a group of elite student-athletes who could develop their dual career under normal conditions, both in pre-Olympic years. It was noteworthy that the student-athletes from the COVID-19 group spent a higher number of hours studying than those from the control group, composed of elite athletes in a normal pre-Olympic year. There was no difference in the number of training sessions or training hours per week between groups, maybe because during the lockdown period caused by COVID-19, the training sessions were performed at home with the aim to keep a physical conditioning routine (Andreato et al., 2020). Previous studies have pointed out that the major barrier Olympic athletes encounter to complete a university degree is time management, since they need to spend over 40 h a week on duties derived from studies and sport (Subijana et al., 2015). This barrier becomes even greater if athletes compete at the international level (Fuchs et al., 2016), like in the present study. One factor that affects the balance between the time spent on training and studying is directly related to the distance from the student-athlete’s home to the training venue or university (Condello et al., 2019). The lockdown has allowed for increased time availability, since these transfers were not needed anymore. This has allowed student-athletes to increase studying hours, without negatively affecting the number of training hours. From the above, it can be hypothesized that, under normal circumstances, switching to online university studies might increase success in the dual career and this is a key aspect to consider when planning dual-career programs in the future. It would allow athletes to better manage their agenda and to reduce their transfer time, thus solving one of the major and most stressful issues for them, which is lesson attendance, especially during competition periods (Gavala-González et al., 2019).

Nonetheless, the significant increase in studying hours has not led to student-athletes’ identity reconfiguration, since they still perceive themselves more as athletes than as students. This result is in keeping with previous studies (Sánchez-Pato et al., 2017; Gavala-González et al., 2019). This could be due to the fact that the study sample was fully composed of international-level elite athletes, which could reinforce their identity as athletes (Lupo et al., 2015).

Another relevant finding was the increase in the perceived benefits of the sport career on the academic career for COVID-19 athletes, compared with a group of Olympic athletes in a normal pre-Olympic year. It is important to highlight that the majority of them obtained university scholarships, thanks to their status of elite athletes. According to previous studies, these athletes are aware of this fact, which could partially compensate the reduced time spent with family and friends, due to the high demands in order to perform well in both sport and academic fields (Gavala-González et al., 2019). The change observed in this item in the COVID-19 group could be due to the fact that elite athletes have become aware during the lockdown that university studies are a means and not a barrier to develop their life projects, and as a consequence, a greater adherence and perceived importance from athletes of dual-career programs may be expected after the COVID-19 pandemic. In this regard, a student-athlete’s priorities are highly determined by the circumstances of their ecosystem (Aquilina, 2009). In contrast to the normative concept of enrolling at university because of social pressure, student-athletes of the COVID-19 group self-realized the importance of holding a university degree for their future after retirement from sport (Jordana et al., 2017; Gavala-González et al., 2019) and that this is a possibility they can only access thanks to their status of elite student-athletes (Debois et al., 2015). In fact, previous studies have stated that motivated athletes are able to combine their academic and sport careers in an optimal manner (Lupo et al., 2015), both dimensions being equally relevant within their identity (O’Neill et al., 2013). Nevertheless, motivations may differ depending on the support structures available to athletes during their dual career (Kerstajn et al., 2018), making further research needed on this topic.

Another noteworthy result was that student-athletes did not consider it easy to achieve balance between their sport and academic careers, in keeping with previous studies (Kristiansen, 2017). Furthermore, there were different opinions about whether the academic career interferes with sport performance. It was much more obvious for student-athletes that sport performance interfered with their academic performance. Both groups presented the same trend in these variables despite the fact that the COVID-19 group dedicated longer time to their academic development. A possible explanation to this would be related to the influence of external factors on student-athletes’ dual career (Guidotti et al., 2015; Kerstajn et al., 2018). Previous studies have revealed that the coach’s role acquires key relevance during student-athletes’ adulthood, being the external factor with the greatest influence on their decision-making and priority setting (Wylleman and Lavallee, 2004). Actually, coaches are usually reluctant to their athletes spending time on studies, in spite of the official rhetoric (Ronkainen et al., 2018). The absence of the coach during the lockdown may have modified their capacity of influencing these factors. Based on this, interventions with coaches would be needed so that they become a positive influence on elite student-athletes’ dual career. This is an important topic to be considered in future research.

COVID-19 has had a huge impact on lifestyle worldwide, and student-athletes have not been an exception. Nevertheless, in general, athletes of the COVID-19 group did not encounter any additional barriers that could hinder success in their dual career, apart from the traditional ones (Subijana et al., 2015). This could be because these athletes were already enrolled on a long-term dual-career program, whose development is guaranteed by the university and the Spanish Olympic Committee. This program has kept running with the required adaptations (Conferencia General de Poliìtica Universitaria, 2020), i.e., face-to-face lessons were suspended and teaching and evaluation was adapted to remote or online mode (Odriozola-González et al., 2020). Furthermore, as the Spanish government decided that universities would autonomously manage their official degrees and master’s programs (Conferencia General de Poliìtica Universitaria, 2020), the university where the participants came from decided to teach the 100% of hours of the face-to-face degrees and master’s virtually, while the online studies continue in the same way, with the sole adaptation to the evaluation to online mode (Universidad Catoìlica San Antonio, 2020). This suggests that the existence of formal support structures is an irreplaceable aspect in the dual career, especially in exceptional situations, and they are expected to be a more successful strategy than the laissez-faire/non-formal models applied in other contexts (Aquilina and Henry, 2019). In fact, student-athletes showed positive perception of some of the characteristics that are inherent to a formal dual-career program, such as the flexible curriculum, sport monitoring of the dual career by the university, the role of the sport tutor, and the services provided by the sport tutor (Isidori, 2016; Sánchez-Pato et al., 2017). Previous studies have already detected that the lack of these features hinders success in the dual career (Fuchs et al., 2016; Gavala-González et al., 2019). According to these findings, it should be proposed that future dual-career programs be based on formal structures and established protocols that help to successfully develop the two areas that converge in the dual career.

The results of the present study revealed improved perception of online learning by the COVID-19 group. Previous studies had already reported that student-athletes prefer online education to the traditional methodology (Tsiatsos et al., 2018), maybe because it allows them more flexibility in their time management, which is the major barrier encountered during the dual career (Subijana et al., 2015). Another aspect that could have influenced this perception is the evolution that online learning resources have experienced in the last few years (Wieman and Gilbert, 2014; Camus et al., 2016). This probably led to a better perception of the learning tools most commonly used by students in online learning (Sánchez-Pato et al., 2017). Based upon these data, the importance of connecting dual-career programs with innovation in education and keeping them up to date must be emphasized in the future.

In accordance with this, student-athletes from the COVID-19 group considered videoconferencing and online tasks to be more important learning resources than the control group, while there were no differences as regards other tools like fora, chat, Facebook, or Twitter. Previous studies have already suggested that students enrolled on online learning programs may better appreciate those tools that are directly related to achievement goal orientation, like the former ones (Dumford and Miller, 2018). Another possible explanation is that during the lockdown caused by COVID-19, the use of the necessary tools in order to turn face-to-face learning into online learning has increased at university (Chaka, 2020), making students who have a positive perception of these resources to use them more. In this line, online tasks were the virtual campus tool that allowed students to submit essays, reports, and similar projects. Through this tool, lecturers can evaluate the assignments and give feedback. During the COVID-19 pandemic, it has been the official way to submit all the works (Universidad Catoìlica San Antonio, 2020), which may explain that students have used it more times as a learning tool. Meanwhile, videoconferencing was the tool provided by the university that allowed students to attend 100% of the theoretical and practical lessons during the lockdown. While students from online modalities used it before the COVID-19 pandemic, students from face-to-face learning had never used it (Universidad Catoìlica San Antonio, 2020), which can explain differences between groups. On the other hand, fora is a tool that aims to create a space of discussion between lecturers and students on a specific topic. This tool was not used in face-to-face studies, while it is optional in virtual ones (Universidad Catoìlica San Antonio, 2020), which can explain why the majority of both groups have never used it. Chats helped students to establish contact between them (Universidad Catoìlica San Antonio, 2020); however, according to the present findings, its use was marginal. This could be a consequence of the students’ preference for the use of smartphones for communication in university environments (Gasaymeh, 2017). In line with the latter, there is not much use of Twitter and Facebook in the current high education context, although previous studies have reported that a pedagogical use of this kind of tools can increase the students’ motivation, learning climate, and academic achievement (Calderón et al., 2019). Further dual-career programs may include this kind of tools in learnings in order to analyze differences in student-athletes’ perception.

This has been the first general emergency experience in the lives of the student-athletes of the COVID-19 group, possibly causing alterations to their emotional status and affecting their decision-making (Shigemura et al., 2020). Consequently, a change of trend has been observed as regards professional expectations after completing their studies, compared with the group of student-athletes in a normal pre-Olympic year. Thus, a lower percentage of COVID-19 athletes intended to continue with their athletic career after completing their studies. This could be due to a change in the student-athletes’ life project. Under normal circumstances, they would have relied more on sport as their main professional activity, but now, in a scenario full of uncertainty, they see their income decrease (Jiménez-Gutiérrez et al., 2020) and, consequently, go for their university career as an instrumental means for life. Nonetheless, the definition of an athlete’s professional path and vocation needs a complex multifactor process (Álvarez-Pérez and López-Aguilar, 2017), which may explain the lack of differences in the rest of the variables regarding professional aspects. This is an important issue for future research.

One interesting finding is that 46.2–55.6% of student-athletes of the current study considered that they were amateur, while 40.3–50.0% considered that they are semiprofessional, although they were high-level athletes, with participations in international competitions, and they had participated in the previous OG or they were eligible to participate in the upcoming OG. The Spanish Sport Law (Jefatura del Estado, 1990) established that only the first and second divisions of the Spanish soccer league and the first division of the Spanish basketball and handball leagues are professional leagues. As a consequence, clubs involve in these competitions act as companies and their players are considered as workers at all levels (salaries, legal rights and duties, etc.) (Martínez-Lemos, 2015). On the contrary, Spanish athletes have difficulty to devote themselves professionally to sport out of these modalities, which is one of the main perceived barriers in order to achieve success in dual career (Subijana et al., 2015). In this line, a high percentage of the student-athletes of the current study were doing some kind of remunerated work in addition to being athletes and students (34.7–38.5%), supporting that athletes’ autoperception is influenced by the fact of being able to live autonomously from sport (North and Lavallee, 2004; Subijana et al., 2015). However, more studies are needed on the relationship between salary and athletes’ self-perception as professional athletes.

Concerning the study limitations, although the questionnaire was found to be a valid and reliable assessment instrument (Sánchez-Pato et al., 2016), it could be interesting to complete the findings in conjunction with other quantitative and quality methods (Kader, 1994). Another limitation was that the current study did not analyze the differences in the student-athletes’ perception of the dual career with a longitudinal design, but with a transversal design involving student-athletes in both pre-Olympic years, one during the lockdown caused by the COVID-19 pandemic and another one under normal conditions. Future studies need to analyze if the changes in the Spanish student-athletes’ perception of the dual career as a consequence of the COVID-19 still remain once the pandemic ends and they can return to their normal lives.

## Conclusion

Student-athletes of the COVID 19-group show adaptations with regard to the organization of their studies and the importance they give to them and to the services provided by dual-career programs, compared with student-athletes from an ordinary pre-Olympic year. In general, student-athletes’ perception of the dual career is very positive. Consequently, student-athletes’ perception allows for reconsideration of the implementation of the dual career under the current circumstances and, especially, in the post-pandemic situation.

## Data Availability Statement

The datasets generated and analyzed for this study can be found in Supplementary Material and at INVESOCIAL database (Fundación Católica de San Antonio, address: Avda. de los Jerónimos de Guadalupe 30107, Murcia, email: investigación@ucam.edu).

## Ethics Statement

The studies involving human participants were reviewed and approved by Ethics Committee. Catholic University of Murcia, Murcia, Spain. The patients/participants provided their written informed consent to participate in this study.

## Author Contributions

LA-C, AL-A, and AS-P conceptualized the study. LA-C, RV-C, JG-R, LM, AL-A, and AS-P designed the study. RV-C and LM carried out the statistical analysis. LA-C, JG-R, AL-A, and AS-P recruited the participants. JG-R and LM collected the data. RV-C organized the database. LA-C, RV-C, JG-R, LM, AL-A, and AS-P wrote the first manuscript draft and the final manuscript draft, conducted the English proofreading, and reviewed and edited the final version of the manuscript. All authors contributed to the manuscript revision and approved the final version.

## Conflict of Interest

The authors declare that the research was conducted in the absence of any commercial or financial relationships that could be construed as a potential conflict of interest.

## Abbreviations

COVID-19: coronavirus disease 2019
OG: Olympic Games.
